# Hispidol Regulates Behavioral Responses to Ethanol through Modulation of BK Channels: A Novel Candidate for the Treatment of Alcohol Use Disorder

**DOI:** 10.3390/molecules29194531

**Published:** 2024-09-24

**Authors:** Wooin Yang, Hee Jae Goh, Young Taek Han, Myon-Hee Lee, Dong Seok Cha

**Affiliations:** 1 College of Pharmacy, Woosuk University, Wanju 55338, Republic of Korea; 2 College of Pharmacy, Dankook University, Cheonan 31116, Republic of Korea; 3 Division of Hematology/Oncology, Department of Internal Medicine, Brody School of Medicine at East Carolina University, Greenville, NC 27834, USA

**Keywords:** alcohol use disorder, BK channel, ethanol intoxication, hispidol, withdrawal

## Abstract

Alcohol use disorder (AUD) is the most common substance use disorder and poses a significant global health challenge. Despite pharmacological advances, no single drug effectively treats all AUD patients. This study explores the protective potential of hispidol, a 6,4′-dihydroxyaurone, for AUD using the *Caenorhabditis elegans* model system. Our findings demonstrate that hispidol-fed worms exhibited more pronounced impairments in thrashes, locomotory speed, and bending amplitude, indicating that hispidol exacerbated the detrimental effects of acute ethanol exposure. However, hispidol significantly improved ethanol withdrawal behaviors, such as locomotory speed and chemotaxis performance. These beneficial effects were absent in slo-1 worms (the ortholog of mammalian α-subunit of BK channel) but were restored with the *slo-1*(+) or *hslo*(+) transgene, suggesting the involvement of BK channel activity. Additionally, hispidol increased fluorescence intensity and puncta in the motor neurons of slo-1::mCherry-tagged worms, indicating enhanced BK channel expression and clustering. Notably, hispidol did not alter internal ethanol concentrations, suggesting that its action is independent of ethanol metabolism. In the mouse models, hispidol treatment also demonstrated anxiolytic activity against ethanol withdrawal. Overall, these findings suggest hispidol as a promising candidate for targeting the BK channel in AUD treatment.

## 1. Introduction

Alcohol is the most consumed addictive substance worldwide. Inappropriate alcohol use can lead to alcohol use disorder (AUD), characterized by an inability to control drinking despite adverse consequences. Alarmingly, over 3 million people die annually due to alcohol-related issues, with the high prevalence of AUD being a major contributor [1]. Despite decades of effort, only three drugs—disulfiram, naltrexone, and acamprosate—have received FDA approval for AUD treatment. Recently, several promising candidates for AUD treatment have emerged, targeting key pathways involved in alcohol consumption, craving, and withdrawal. These include corticotropin-releasing factor-1 (CRF1) antagonists, α1-adrenoceptor antagonists, glycine reuptake inhibitors, phosphodiesterase inhibitors, PPAR agonists, NMDA receptor antagonists, and oxytocin receptor agonists. However, despite their potential, many of these pharmacotherapies have shown limited efficacy and are associated with various adverse effects [2]. Therefore, there is an urgent need to uncover neurobiological targets and develop novel agents for AUD treatment.

The large-conductance, voltage- and calcium-dependent potassium channel (BK channel) is activated by membrane depolarization and increased intracellular calcium, leading to hyperpolarization and precise regulation of calcium-dependent processes such as muscle excitation, neurotransmitter release, and hormone secretion [3]. Extensive research using forward and reverse genetic approaches across various animal models and humans has identified the BK channel as a highly conserved regulator of ethanol-induced behavioral sensitization [4]. Clinically relevant concentrations of ethanol (10~100 mM) are sufficient to activate the BK channel, leading to neuronal hyperpolarization and reduced excitability across diverse phyla [5]. In invertebrates, alcohol-induced behaviors, such as acute intoxication and withdrawal, can be altered by genetic manipulation of BK channel. Correlations between BK channel activity and alcohol-related behaviors have also been observed in rodents and humans [6,7,8]. Thus, targeting the BK channel is an attractive strategy for developing novel AUD treatments.

**Figure 1:**
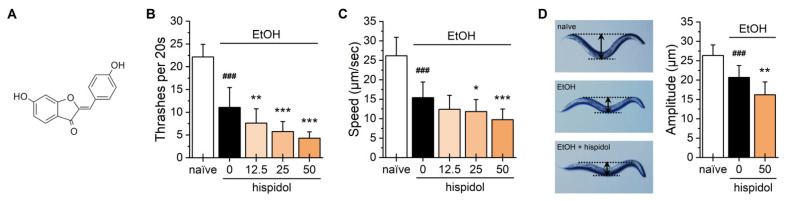
Effects of hispidol on ethanol-induced acute behaviors in *C. elegans*. (**A**) Chemical structure of hispidol; Age-synchronized worms were exposed to 300 mM ethanol for 10 min, and ethanol-induced behavioral phenotypes, including (**B**) thrashing, (**C**) locomotion speed, and (**D**) body amplitude were observed. Data are presented as the mean ± S.D., with results obtained from three independent experiments. Statistical significance is indicated as follows: ^###^ *p* < 0.001 compared with naïve animals; * *p* < 0.05, ** *p* < 0.01, *** *p* < 0.001 compared with ethanol-exposed animals.

Hispidol, a 6,4′-dihydroxyaurone, is a structural isomer of flavone (Figure 1A). This yellow compound has been isolated from *Glycine max*, *Lygos raetam*, and *Medicago truncatula*. Previous studies have unveiled the antimicrobial, anti-inflammatory, antidepressant, and longevity properties of hispidol [9,10,11,12]. Furthermore, hispidol has been shown to inhibit key enzymes in metabolism such as glucosidase, tyrosinase, and monoamine oxidase A [13,14,15]. Given its diverse bioactivity, we evaluated the efficacy of hispidol in modulating alcohol-induced behaviors using the *Caenorhabditis elegans* model system—an excellent platform for studying drug addiction [16].

## 2. Results

### 2.1. Hispidol Amplifies Acute Ethanol-Intoxicating Behaviors

To test the effect of hispidol on ethanol-induced acute responses, test worms were incubated on a 300 mM ethanol (resulting in approximately 25 mM internal ethanol levels in worms) plate for 10 min, and their behavioral phenotypes were observed. We found that exposure to ethanol significantly reduced thrashing rate and velocity. Interestingly, hispidol treatment dose-dependently amplified these ethanol-induced sedative behavioral phenotypes (Figure 1B,C). In addition, ethanol decreased the amplitude of body bends in worms, with hispidol supplementation leading to almost flattened body bends in ethanol-exposed worms (Figure 1D). These results suggest that hispidol may have the potential to reduce the amount of alcohol consumed to reach intoxication, which could contribute to AUD treatment.

### 2.2. Hispidol Ameliorates Ethanol-Withdrawal Behaviors

**Figure 2:**
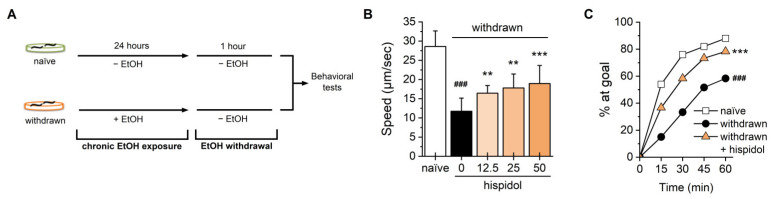
Effects of hispidol on ethanol withdrawal-induced behaviors in *C. elegans*. (**A**) Age-synchronized worms were exposed to 150 mM ethanol for 24 h and subsequently transferred to fresh plates for a 1-h withdrawal period; (**B**) Locomotion speed of withdrawn worms was assessed under a dissecting microscope; (**C**) The chemotactic ability of withdrawn worms toward the attractant OP50 was monitored every 15 min for 1 h. Statistical significance is indicated as follows: ^###^ *p* < 0.001 compared with naïve animals; ** *p* < 0.01 and *** *p* < 0.001 compared with ethanol-exposed worms.

We then investigated hispidol’s effects on ethanol withdrawal by observing behavioral changes 1 h after withdrawal in chronically ethanol-exposed worms. As shown in Figure 2B, the withdrawn worms exhibited slower crawling speed compared to ethanol-untreated controls. Hispidol treatment improved this ethanol withdrawal-induced behavioral deficit in a dose-dependent manner (Figure 2B). In the chemoattractant race assay, withdrawn worms also displayed a significant reduction in the chemotactic response to food, which was successfully ameliorated by hispidol (Figure 2C). These findings suggest that hispidol may have therapeutic potential for ethanol withdrawal-induced behavioral impairment.

### 2.3. BK Channel Modulation Is Required for the Hispidol-Mediated Changes in Ethanol-Induced Behaviors

**Figure 3:**
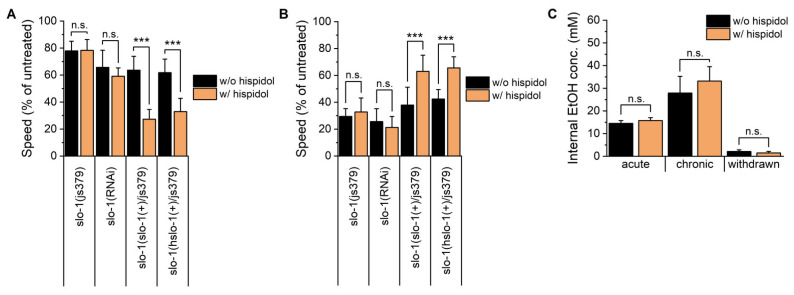
Involvement of BK channel modulation in hispidol-mediated alterations of ethanol-induced behaviors. (**A**) Age-synchronized *slo-1(js379)*, *slo-1(RNAi)*, *slo-1(+/js379)*, and *slo-1(hslo-1+/js379)* worms were exposed to 300 mM ethanol for 10 min, and their locomotion speed was measured; (**B**) Locomotion speed of *slo-1(js379)*, *slo-1(RNAi)*, *slo-1(+/js379)*, and *slo-1(hslo-1+/js379)* worms was assessed following ethanol withdrawal; (**C**) Internal ethanol concentration in wild-type worms was spectrophotometrically analyzed at three different time points: after 10 min of ethanol exposure (acute), after 24 h of ethanol exposure (chronic), and after an additional 1-h withdrawal following 24 h of ethanol exposure). Statistical significance is indicated as follows: *** *p* < 0.001 compared with hispidol-untreated worms; n.s. not significant.

Given the importance of BK channel in behavioral effects of ethanol, we tested whether hispidol influences BK channel to modulate ethanol-induced responses during acute intoxication and withdrawal [4]. We measured the velocity of *slo-1* (js379) worms and wild-type worms with RNAi of *slo-1* after acute ethanol exposure and ethanol-withdrawal. Interestingly, hispidol-mediated behavioral modifications observed in wild-type worms completely disappeared in both *slo-1* null mutants and *slo-1* (RNAi) treated worms (Figure 3A,B). We also found that both the *slo-1*(+) (vxEx345) and human *slo-1*(+) (vxEx339) transgenes restored hispidol’s effects in *slo-1* null mutants (Figure 3A,B). These results confirm that hispidol’s actions involve slo-1 modulation and suggest it may also interact with human slo-1. Furthermore, hispidol’s effects were not due to altered ethanol metabolism, as internal ethanol concentrations remained similar between treated and untreated worms (Figure 3C).

### 2.4. Hispidol Activates BK Channel via Clustering Regulation

**Figure 4:**
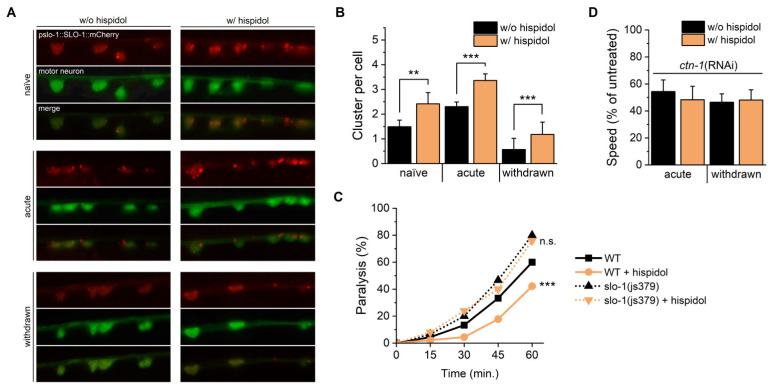
Effects of hispidol on BK channel clustering. (**A**) Expression patterns of mCherry in JPS572 (vxEx345 [slo-1p::slo-1(+)::mCherry::unc-54 3′UTR + myo-2p::mCherry]) worms, captured at 100× magnification using fluorescence microscopy; (**B**) Quantification of slo-1 puncta, representing clustered BK channels in cholinergic neurons; (**C**) Carbofuran-induced paralysis monitored every 15 min in wild-type and *slo-1*(*js379*) worms; (**D**) Locomotion speed of *ctn-1(RNAi)* worms observed after 10 min of ethanol exposure and following withdrawal induction. Statistical significance is indicated as follows: ** *p* < 0.01, *** *p* < 0.001 compared with hispidol-untreated worms; n.s., not significant.

The critical role of slo-1 clustering in ethanol-mediated responses is well documented. Since modifications in slo-1 function in motor neurons are crucial for ethanol-induced behaviors, we examined hispidol’s effects on slo-1 clustering in cholinergic neurons using transgenic mutants carrying vsIs48 transgene [17] As can be seen in Figure 4A,B, acute ethanol exposure increased red-fluorescent puncta in the green fluorescent overlapped region, and hispidol treatment accelerated this cholinergic neuron specific puncta formation. Consistent with previous findings, puncta were reduced during withdrawal, while hispidol treatment restored this decrease in puncta (Figure 4A,B) [18]. Notably, hispidol also enhanced puncta formation in ethanol-naïve worms. BK channels prevent excessive depolarization to regulate neurotransmitter release, but in *slo-1* mutants, their dysfunction leads to uncontrolled acetylcholine release, increasing sensitivity to acetylcholinesterase inhibitors like carbofuran, resulting in rapid paralysis due to acetylcholine buildup at synapses [19]. In this study, carbofuran-induced paralysis was attenuated by hispidol in wild-type worms but not in *slo-1* (js379) worms (Figure 4C). These results suggest that hispidol’s protective role depends on functional BK channels in cholinergic neurons and may stabilize cholinergic signaling by modulating BK channel activity independently of ethanol. Given the crucial role of the cytoskeletal protein ctn-1, an α-catulin ortholog, in slo-1 localization, we further tested the requirement of ctn-1 for hispidol-mediated clustering acceleration [20,21]. We found that ctn-1 (RNAi) worms showed resistance to acute intoxication and increased withdrawal severity, and hispidol had no significant impact on either acute response or withdrawal (Figure 4D) [20]. This suggests that hispidol regulates slo-1 functions through ctn-1-dependent mechanism.

### 2.5. Hispidol Improves Anxiety-Like Behaviors in Ethanol-Withdrawan Mice

**Figure 5:**
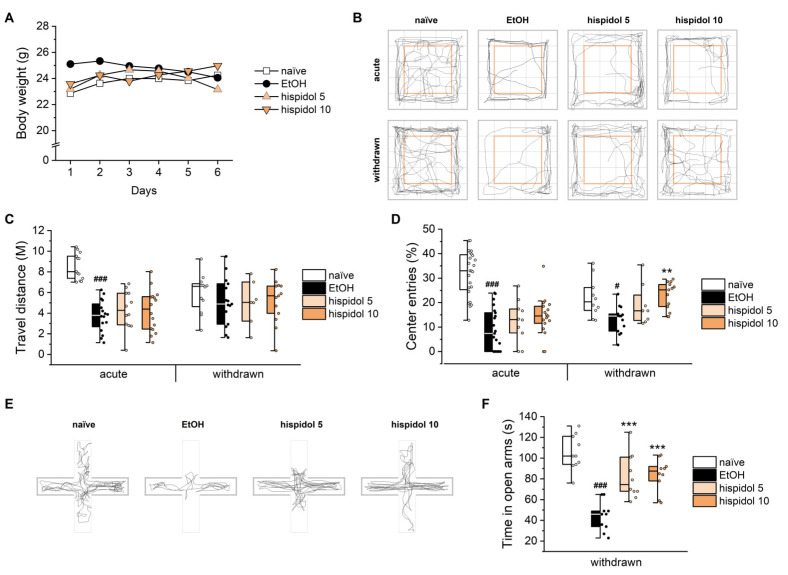
Effects of hispidol on ethanol-induced behaviors in mice. (**A**) Body weights of C57BL/6 mice monitored over seven days during a series of ethanol intoxications; (**B**) Motion trajectories recorded during the open field test; (**C**) Total distance traveled and (**D**) entries of center zone by mice in the open field test; (**E**) Motion trajectories recorded during the elevated plus maze test; (**F**) Time spent in the open arms in the elevated plus maze test. Statistical significance is indicated as follows: ^#^ *p* < 0.05 and ^###^ *p* < 0.001 compared with naïve animals; ** *p* < 0.01 and *** *p* < 0.001 compared with ethanol-exposed worms.

To determine whether the efficacy observed in worms extends to vertebrates, we evaluated hispidol in a mouse AUD model using open field test (OFT) and elevated plus maze test (EPMT). As shown in Figure 5A, hispidol-treated mice showed no significant changes in body weight over the 7-day ethanol exposure period. In the OFT, hispidol failed to affect the total distance traveled under both acute intoxicated and withdrawn conditions (Figure 5B,C). However, the trajectory of withdrawn mice indicated that hispidol treatment alleviated the withdrawal-induced decrease in the center ratio, suggesting reduced anxiety-like behavior (Figure 5B,D) [22]. In the EPMT, hispidol counteracted the reduced time spent in open arms by withdrawn mice (Figure 5E,F). Conclusively, these results suggest that hispidol exerts anxiolytic effects in withdrawn mice that are independent of overall locomotion. Given that the BK channel facilitates rapid neuronal repolarization and attenuates glutamate release, thereby reducing excitability, it is plausible that hispidol-mediated BK channel activation may play a role in anxiety control [23,24]. However, further research is needed to determine whether BK channel modulation is solely responsible for these effects.

## 3. Discussion

Genetic screening in *C. elegans* based on behavioral responses to ethanol identified the *slo-1* gene, encoding the α-subunit of BK channel, as crucial for intoxicating effects of ethanol [25]. A similar level of behavioral depression was observed in both *slo-1* gain-of-function mutants and ethanol-treated wild-type worms, suggesting that BK channel activation is crucial for ethanol-induced acute response [17].

Similarly, additional research in mammals, including rodents and humans, has supported this initial finding in worms [4]. This conserved function of the BK channel across species makes it a promising target for AUD therapy [5,26,27]. In this study, hispidol was found to activate BK channel independently of ethanol, enhancing the responses to acute intoxication and resulting in reduced behavioral activity. This hispidol-mediated enhancement of acute ethanol responses suggests that intoxication could be achieved with lower alcohol consumption when hispidol is present, potentially reducing excessive drinking and providing therapeutic benefits for AUD. Although we did not observe any significant changes in locomotion performance in hispidol-fed mice during the ethanol exposure period, further research is needed to determine whether this approach would mitigate or exacerbate the harmful effects of alcohol in higher organisms.

Long-term exposure to ethanol is believed to induce adaptive responses of the nervous system [28]. These homeostatic changes include the down-regulation of BK channel function to compensate for ethanol-induced excessive and prolonged activation of the channel. Studies in mammals have shown that chronic ethanol exposure lowers BK channel expression and raises the abundance of BK channel isoforms insensitive to ethanol [27]. Similarly, the severity of withdrawal is bi-directionally related to slo-1 function of motor neurons in *C. elegans* [18]. Hence, enhanced BK channel activity can improve withdrawal-induced behavioral impairments, while inhibition of the channel can worsen them. Our study showed that hispidol improved withdrawal-induced behavioral deficits in worms through slo-1 modulation in cholinergic motor neurons, consistent with its effects during acute intoxication.

Previous studies identified several BK channel regulatory factors, including lipid microenvironment, channel clustering and trafficking, β-subunit-mediated modulation, and post-translational regulation by miR9 under ethanol exposure conditions [29]. In this study, we focused on the role of slo-1 clustering in the bioactivity of hispidol because ethanol-mediated responses to both acute intoxication and withdrawal are closely linked with slo-1 localization. Specifically, the interaction of BK channels with the actin cytoskeleton and trafficking proteins is crucial for their precise localization in neuronal membranes, which in turn affects cellular excitability and response to ethanol. Our results showed that hispidol treatment accelerates ethanol-induced slo-1 clustering, potentially through a ctn-1 dependent mechanism. This suggests that hispidol may enhance BK channel clustering by stabilizing its interaction with cytoskeletal elements, particularly the ctn-1 protein, which is crucial for presynaptic slo-1 localization and ethanol sensitivity, rather than through direct interaction with channel proteins [20,21]. Given that BK channels function in either diffused or clustered state in worms and mammals, targeting channel localization may offer a more effective treatment strategy than indiscriminately targeting the channel.

Can these efficacies of hispidol observed in invertebrates be replicated in mammals, particularly humans? In worms and flies, BK channel involvement in ethanol-induced behaviors appears straightforward, while the mechanism in mammals is more complex. Ethanol exposure in mammals induces different responses in BK channels depending on tissue type and their location of cells. For example, ethanol potentiates neuronal (motor neurons but not sensory neurons) BK channels while inhibiting aortic BK channels [30]. In neurons, BK channels at the nerve-terminal are activated by ethanol, but cell-body channels are not [31]. Moreover, BK channel activation in mammals can be regulated directly or indirectly by factors such as calcium levels, miR9, and specific β subunits. Additionally, given the genetic variance among individuals, drugs targeting regulatory factors may not be equally effective for all AUD patients. Therefore, promising BK channel modulators for AUD treatment should: (i) act tissue-specifically, (ii) target specific neuronal compartments, and (iii) avoid disrupting regulatory factors. While hispidol was found to reduce withdrawal-induced anxiety, at least in mice, additional studies are required to evaluate whether hispidol meets these criteria and remains effective in humans. In conclusion, hispidol amplifies ethanol’s acute effects while alleviating withdrawal symptoms through BK channel modulation, suggesting its potential as a therapeutic candidate for AUD.

## 4. Materials and Methods

### 4.1. Preparation of Hispidol

Hispidol was synthesized in accordance with our previous research, and we confirmed that the NMR spectra data of the purified final compound matched the data published earlier [11,32]. The synthesized hispidol was stored at a temperature of −20 °C. For the preparation of plates containing hispidol, the stock solution in dimethyl sulfoxide (DMSO) was appropriately diluted with M9 buffer and added to autoclaved nematode growth medium (NGM) agar plates. A final concentration of DMSO was maintained at less than 0.1% (*v*/*v*) under all experimental conditions.

### 4.2. C. elegans Methods

#### 4.2.1. *C. elegans* Strains and Maintenance

The following strains were utilized in the present study: N2 (wild-type), NM1968 slo-1(js379), JPS572 slo-1(js379); vsIs48 [unc-17::GFP]; vxEx345 [slo-1p::slo-1(+)::mCherry::unc-54 3′UTR + myo-2p::mCherry], and JPS574 slo-1(js379); vsIs48 [unc-17::GFP]; vxEx339 [slo-1p::hslo(+)::mCherry::unc-54 3′UTR + myo-2p::mCherry]. These strains were kindly provided by the Caenorhabditis Genetic Center (CGC; University of Minnesota, Minneapolis, MN). The worms were cultured on NGM agar plates seeded with Escherichia coli OP50 and maintained at a temperature of 20 °C. To ensure consistent testing conditions, age synchronization of the worms was performed by isolating embryos, thereby minimizing variations arising from differences in worm age.

#### 4.2.2. RNA Interference

RNA interference (RNAi) by feeding was performed as previously described [33]. In brief, slo-1 and ctn-1 RNAi bacteria were cultured in LB medium supplemented with 50 μg/mL ampicillin for 12 to 16 h. The cultured bacteria were then seeded onto NGM agar plates containing 1 mM IPTG and subsequently incubated at room temperature for 24 h. L1 stage worms were transferred onto the seeded plates and allowed to feed for four days. The resulting first-day adult worms were collected and subjected to subsequent behavioral assays.

#### 4.2.3. Behavioral Assays

Ethanol plates with concentrations of 300 mM and 150 mM were prepared by adding ethanol beneath the NGM agar plates. The plates were immediately sealed with parafilm and incubated at room temperature for 2 h to facilitate ethanol absorption into the agar. Age-synchronized adult day 1 worms were then transferred to a 300 mM ethanol plate to induce acute intoxication. Following a 10-min exposure to ethanol, their behavioral phenotypes, including thrashing, body amplitude, crawling speed, and egg laying, were observed for 1 min. To induce withdrawal behavior, age-synchronized adult day 1 worms were placed on a 150 mM ethanol plate for 24 h. Then, the ethanol-treated worms were incubated for an additional hour on a fresh plate before being subjected to subsequent experiments (Figure 2A). The crawling speed of withdrawn worms was measured for 1 min, and their chemotaxis performance was assessed as described previously [18]. All locomotory features were recorded and analyzed using Tierpsy tracker and Image J software 1.54d. Carbofuran-induced paralysis assay was performed as described previously with minor changes [34]. Briefly, age-synchronized adult day 1 worms were transferred to a plate containing 1 mM carbofuran. The paralyzed worms, characterized by the absence of body or pharyngeal movement, were counted at 15-min intervals.

#### 4.2.4. Analysis of Internal Ethanol Concentration

The internal ethanol concentration was determined following the manufacturer’s instructions provided with the Alcohol Reagent Kit from Pointe Scientific (Lincoln Park, MI, USA). Briefly, a total of 400 worms was collected and washed twice with M9 buffer. The worm pellet was subsequently resuspended in a homogenization buffer containing 10 mM Tris-HCl, 150 mM NaCl, 0.1 mM EDTA (pH 7.5), and then homogenized on ice. The resulting worm homogenate was combined with the alcohol reagent and incubated for 5 min at 30 °C. To terminate the reaction, the test tube was promptly placed on ice. The concentration of ethanol in the worm homogenates was measured spectrophotometrically at a wavelength of 340 nm.

#### 4.2.5. Fluorescence Microscopy and Visualization

To detect fluorescence signals, the test worms were immobilized using 10% sodium azide and mounted onto 2% agarose pads. Subsequently, fluorescent images were captured using a Nikon Eclipse Ni-u fluorescence microscope (Nikon, Tokyo, Japan) at a magnification of 100×. The acquired images were then analyzed using ImageJ software to investigate the expression and clustering of slo-1.

### 4.3. Mouse Methods

#### 4.3.1. Subjects

Male C57BL/6 mice (6 weeks old, weighing 20–23 g, *n* = 50) were obtained from Samtako Bio Korea. The mice had access to tap water and food ad libitum and were housed under standard conditions (temperature: 21–24 °C; humidity: 45–60%; light/dark cycle: 12–12 h). After a 7-day acclimation period, the mice were randomly assigned to five groups, each consisting of six mice. All experimental procedures were conducted in accordance with the ethical regulations of the Animal Experiment Ethics Committee of Woosuk University, approved under protocol number WS-2023-19.

#### 4.3.2. Open Field Test

The Open Field Test (OFT) was performed 5 min after a single intragastric (i.g.) injection of ethanol (4 g/kg) for acute ethanol treatment. To induce dependence, ethanol was administered once daily for 7 days. The OFT was conducted 6 h after the last administration of ethanol to evaluate withdrawal symptoms. Thirty minutes before the ethanol injection, all mice were intraperitoneally administered hispidol (5 or 10 mg/kg) or the vehicle (10% DMSO, 10% saline in Tween80). The arena consisted of a square box (30 cm length × 30 cm width × 30 cm height) lined with black tape. Forward locomotion was evaluated by recording the total distance for 5 min, measuring locomotion when the animal was in a prone position, moving all four paws simultaneously. The frequency of rearing was also measured. Rearing was defined as when the mice stood on their hind legs away from the wall. Mice behaviors were further analyzed based on the total distance traveled in the central area of the open field. The central area was virtually defined as a 36 cm² square occupying the center of the arena. The total distance traveled in this central section is commonly considered a measure of anxiety. Video footage of behavior in the open field and across tests was recorded by a camera affixed above the arena. The arena was cleaned with 70% ethanol between trials, and the data were subsequently analyzed by an observer unaware of the group assignment.

#### 4.3.3. Elevated Plus Maze Test

Similar to the open field test, anxiety-like behaviors of withdrawn mice in the Elevated Plus Maze (EPMT) were evaluated for 5 min. The EPM consisted of two open arms (45 cm × 5 cm) and two closed arms (45 cm length × 5 cm width × 45 cm height) that extended from a common central platform (5 cm × 5 cm), elevated 50 cm above the floor. Briefly, each mouse was gently placed in the center platform facing an open arm and recorded by a video camera affixed above the maze. The time spent in open arms was measured and considered as indices of anxiety. The arena was cleaned with 70% ethanol between trials.

### 4.4. Data Analysis

The statistical analysis of the obtained data was performed using Origin Pro software 2023 (10.0). All data were presented as mean ± standard deviation (S.D.), and the statistical significance of differences among groups was assessed using ANOVA following the Bonferroni post hoc comparisons.

## 5. Conclusions

In this study, we identified hispidol, a 6,4′-dihydroxyaurone, as a potential therapeutic candidate for AUD through its modulation of the BK channel. Our findings in *C. elegans* revealed that hispidol amplified ethanol-induced acute behavioral impairments but significantly improved withdrawal symptoms, effects that were dependent on BK channel activity. In mouse models, hispidol demonstrated anxiolytic effects during withdrawal without altering body weight or overall locomotion. These results indicate that hispidol may modulate ethanol-induced behaviors through BK channels, offering a promising strategy for AUD treatment. However, further preclinical and clinical studies are warranted to fully evaluate hispidol as a novel pharmacotherapy for AUD.

## Data Availability

Most of the data generated in this work is included in the manuscript, and the raw data can be shared upon request from the corresponding author.
